# Tubeimoside I Antagonizes Yoda1-Evoked Piezo1 Channel Activation

**DOI:** 10.3389/fphar.2020.00768

**Published:** 2020-05-25

**Authors:** Silin Liu, Xianmei Pan, Wenbin Cheng, Bo Deng, Yu He, Lei Zhang, Yile Ning, Jing Li

**Affiliations:** ^1^Lingnan Medical Research Center, Guangzhou University of Chinese Medicine, Guangzhou, China; ^2^The First Affiliated Hospital, Guangzhou University of Chinese Medicine, Guangzhou, China; ^3^School of Pharmaceutical Sciences, Guangzhou University of Chinese Medicine, Guangzhou, China

**Keywords:** tubeimoside I, Chinese medicine, Piezo1 channels, inhibitor, Yoda1, vascular pharmacology

## Abstract

Piezo1, a mechanosensitive Ca^2+^-permeable non-selective cationic ion channel protein, is involved in a wide range of biological processes and plays crucial roles in vascular development. However, the pharmacology of this protein is in its infancy. Yoda1, the first specific chemical activator of Piezo1 channels, can activate Piezo1 in absence of mechanical stimulation. Hence, we sought to identify inhibitors of Yoda1 from Traditional Chinese Medicine (TCM). Intracellular Ca^2+^ measurements were conducted in human umbilical vein endothelial cells (HUVECs), HEK 293T cells overexpressing TRPC5 and TRPM2 channels, as well as in CHO K1 cells overexpressing TRPV4 channels. We identified tubeimoside I (TBMS1) as a strong inhibitor of the Yoda1 response and demonstrated its selectivity for the Piezo1 channels. Similarly, Yoda1-induced inhibitory results were obtained in Piezo1 wild-type overexpressed cells, murine liver endothelial cells (MLECs), and macrophages. The physiological responses of TBMS1 were identified by isometric tension, which can inhibit Yoda1 relaxation of aortic rings. Our results demonstrated that TBMS1 can effectively antagonize Yoda1 induced Piezo1 channel activation. This study sheds light on the existence of Yoda1 inhibitors and improves the understanding of vascular pharmacology through Piezo1 channels.

## Introduction

Piezo1 is a mechanically activated ion channel involved in a wide range of biological functions, including touch perception, proprioception, and vascular blood flow (Wu et al., 2017; Zhao et al., 2018). It forms a trimer with a propeller-like structure, with the extracellular regions resembling three distal blades surrounding a central cap (Ge et al., 2015; Guo and MacKinnon, 2017; Saotome et al., 2018; Zhao et al., 2018). Piezo1 is involved in sensing endothelial shear stress, vascular development and remodeling, managing blood pressure, and exercise performance (Coste et al., 2010; Li et al., 2014; Ranade et al., 2014; Retailleau et al., 2015; Rode et al., 2017). Mutations in the human *PIEZO1* gene cause anemia (dehydrated stomatocytosis) and generalized lymphatic dysplasia, consistent with the protein's importance in regulation of erythrocyte volume and epithelial cell homeostasis (Eisenhoffer et al., 2012; Zarychanski et al., 2012; Albuisson et al., 2013; Fotiou et al., 2015; Lukacs et al., 2015; Andolfo et al., 2016; Gudipaty et al., 2017). These observations demonstrate the functional value of Piezo1 channels and their feasibility as a medicinal target.

However, Piezo1 pharmacology is in its infancy. The first potent and specific activator of Piezo1 is Yoda1, a synthetic small molecule, which can activate Piezo1 channel in the absence of mechanical stimuli (Syeda et al., 2015). Subsequently, Jedi was identified as a novel type of chemical activator of Piezo1. Specifically, Jedi appears to activate and modulate Piezo1 by acting on loci along the blade-beam gating pathway distinct from those activated by Yoda1 (Wang et al., 2018). However, the inhibitors of the channel are restricted to generic inhibitors of ion pores, like gadolinium III (Gd^3+^) and ruthenium red (Drew et al., 2002; Coste et al., 2012). The Yoda1 analogue Dooku1 antagonizes the Yoda1-induced response of Piezo1 and aortic relaxation (Evans et al., 2018). Thus, Yoda1 is a key tool for understanding Piezo1 inhibitors.

In the present study, we took advantage of Yoda1 to conduct a screen of 92 different molecules from Traditional Chinese Medicine (TCM), comparing their effects on Piezo1 channels, other channels, and vasoconstriction. Tubeimoside I (TBMS1), a triterpenoid saponin present at high levels in the Chinese herbal medicine Bolbostemma paniculatum (Maxim) Franquet (Cucurbitaceae) (Chinese name “Tu Bei Mu”) (Tang et al., 2015; Yang et al., 2016), stood out as an effective inhibitor of the Yoda1 response with selectivity for the Piezo1 channel. Our findings are a crucial step toward obtaining a better understanding of Piezo1 and developing novel Piezo1 regulators.

## Methods

### Cell Culture

Human umbilical vein endothelial cells (HUVECs) purchased from Promocell (Germany) were maintained in Endothelial Basal Medium 2 (EBM2) supplemented with Bullet Kit (Lonza, Basel, Switzerland) containing growth factors (50 ng·ml^-1^ gentamicin, 10 ng·ml^-1^ VEGF, 1 μg·ml^-1^ hydrocortisone, 5 ng·ml^-1^ human basic FGF, 50 ng·ml^-1^ amphotericin B, and 2% FCS) and 10 μg·ml^-1^ heparin. HUVECs used for experiments were passaged two to six times. For TRPC5- and TRPM2-expressing HEK 293T cells, selection was performed by adding 5 μg·ml^-1^ blasticidin and 400 μg·ml^-1^ zeocin to DMEM (Gibco, USA) supplemented with 10% fetal bovine serum (FBS) and 1% penicillin/streptomycin. For TRPV4-expressing Chinese hamster ovary (CHO) K1 cells, they were maintained in Ham's F12 (Gibco, USA) in the presence of 1mg/ml G418 (Sigma, Shanghai). To induce Tet-dependent gene expression, cells were incubated with 1 μg·ml^-1^ tetracycline for 24 h prior to experiments. Human myeloid leukemia mononuclear cells (THP-1) and a murine monocytic cell line (RAW264.7) were sustained in RPMI-1640 supplemented with 1% penicillin/streptomycin and 10% FBS. All cells were grown at 37°C in a 5% CO_2_ humidified incubator.

Murine liver tissue samples were preserved in cold EBM-2 medium. Endothelial cells were isolated by the CD31 microbead technique. Initially, the tissue was minced using two scalpel blades and resuspended in a dissociation solution composed of 9 ml 0.1% collagenase II, 1 ml 2.5 U·ml^-1^ dispase, 1 µM calcium chloride, and 1 μM magnesium chloride in Hanks Buffer. The tissue-dissociation mix was incubated in a MACSMix Tube Rotator (Miltenyi Biotech) at 37°C for 45 min to provide continuous stirring. At the end of enzymatic digestion, to remove undigested tissue, the sample was passed through 100 μm and 40 µm cell filters. Cells were washed twice in magnetically activated cell sorting (MACS) buffer consisting of phosphate-buffered saline (PBS), 2 mM EDTA, and 0.1% bovine serum albumin (BSA), pH 7.2. The washed pellets were suspended in 20 ml red blood cell lysis buffer containing 0.206 g Tris base and 0.749 g NH_4_Cl in 100 ml PBS (pH 7.2) for 10 min, and then washed for a final time in MACS buffer. Next the pellet was incubated with 200 µl/1 × 10^7^ total cells of dead cell removal paramagnetic microbeads (Miltenyi Biotec) and incubated at room temperature for 15 min. After incubation, the cells were passed through a LS column prepared with 1× Binding Buffer (Miltenyi Biotec) in a magnetic field (MiniMACS Separator, Miltenyi Biotec). The eluate was incubated with 30 µl FcR blocking reagent and 30 µl CD31-conjugated paramagnetic microbeads (Miltenyi Biotec) at 4°C for 15 min. After incubation, the solution was prepared with an MS column and MACS buffer. CD31-positive cells remained in the column, and CD31 negative cells passed through as eluents. CD31-positive cells were washed with warm EBM-2 medium, placed in one well of a 6-well plate coated with 0.1% gelatin, and incubated in a 5% CO_2_ incubator at 37°C. Medium was changed at 12 h, and then every 48 h until conﬂuent.

### Intracellular Ca^2+^ Measurements

TRPC5- and TRPM2-overexpressing HEK 293T cells (Hecquet et al., 2008; Amer et al., 2013; Akbulut et al., 2015) were plated at 90% confluence in poly-D-lysine–coated 96-well plates, and cells of other types were plated in clear 96-well plates. After 24 h, cells were incubated with working solution containing 2 μM Fura-2-AM (Molecular Probes) and 0.01% pluronic acid (Thermo Fisher Scientific) in standard bath solution (SBS) for 1 h at 37°C in an incubator. Whereas Fluo-4AM was used in place of Fura-2AM for recordings of overexpressed TRPV4 activity (Thodeti et al., 2009; Ma et al., 2010; Li et al., 2011). Cells were washed with SBS (130 mM NaCl, 8 mM D-glucose, 5 mM KCl, 10 mM HEPES, 1.5 mM CaCl_2_, 1.2 mM MgCl_2_; titrated to pH = 7.4 with NaOH) twice at room temperature, after which TBMS1 was added; cells were pretreated for 30 min with TBMS1, and this treatment continued during subsequent experimental manipulations. Measurements were carried out on a 96-well fluorescence plate reader (FlexStation; Molecular Devices, Sunnyvale, CA, USA). The change (Δ) in intracellular calcium was calculated based on the ratio of Fura-2 emission (510 nm) intensities excited at 340 and 380 nm and for Fluo-4AM emission (515 nm) excited at 495 nm. Measurements are shown as absolute fluorescence in arbitrary units, with baseline fluorescence defined as zero.

### Piezo1 Plasmid Transfection

For overexpression of GFP-tagged wild-type Piezo1 (Li et al., 2014), HEK 293T cells were transfected with 200 ng plasmid using FuGENE^®^ HD (Promega), and intracellular Ca^2+^ measurements were made after 48 h.

### Animals and Aorta Contraction Studies

All mice (C57BL/6 male mice, 12~16-week-old) used for experiments were housed in individually ventilated cages under the following conditions: temperature, 21°C; humidity, 50~70%, and light/dark cycle, 12 h/12 h. All animal experiments were authorized by the Guangzhou University of Chinese Medicine Animal Ethics Committee. Animals were killed by CO_2_ asphyxiation. The thoracic aorta was dissected and placed directly in ice-cold Krebs solution (125 mM NaCl, 1.5 mM MgSO_4_, 3.8 mM KCl, 0.02 mM EDTA, 25 mM NaHCO_3_, 1.2 mM CaCl_2_, 1.2 mM KH _2_PO_4_, and 8 mM D-glucose, pH 7.4). Connective tissue and fat were carefully removed under an anatomical microscope. One-millimeter segments were mounted in an isometric wire myograph system with two stainless steel wires 40 μm in diameter, bathed in Krebs solution at 37°C, and blistered with 95% O_2_ and 5% CO_2_. The segment was then gradually stretched to its optimal resting tension to 5mN and then balanced for 1 h before the experiment. The aortic ring was stimulated twice with 60 mM high potassium Krebs solution. Before Yoda1 was added, phenylephrine (PE) was applied to contract the vessel. For the TBMS1 experiment, the aortic ring was pretreated with TBMS1 for 30 min before application of PE and Yoda1.

### Immunofluorescence

Cells were fixed with 4% paraformaldehyde for 15 min and permeabilized with 0.1% Triton X-100 for 10 min at room temperature. Non-specific sites were blocked using 2% BSA in PBS for 30 min at room temperature. Cells were then incubated in 1% BSA in PBS containing mouse anti-human CD31/PECAM-1 (clone JC70A; Dako) at 1:500 dilution overnight at 4°C. After washing with PBS, cells were incubated with DyLight 649-conjugated AffiniPure donkey anti-mouse IgG (Jackson Immuno Research Laboratories) for 1 h at room temperature. Cells were mounted with Prolong Gold Antifade Reagent (Invitrogen) and visualized using a Delta Vision deconvolution system (Applied Precision Instruments, Seattle, WA, USA) on an Olympus IX-70 inverted microscope fitted with 360 UPLAN objective (NA 1.35). For F-actin staining, cells were incubated with 1:250 rhodamine phalloidin (Cytoskeleton Inc.) for 30 min at room temperature. Cell nuclei were labeled with DAPI (4,6-diamidino-2-phenylindole) with an excitation wavelength of 350 nm. Images were captured using a confocal microscope (Zeiss LSM 880).

### RT-PCR Assays

Total RNA was extracted using a Tri-reagent protocol followed by DNase I (Ambion) treatment. Reverse transcription (RT) was performed using a high capacity RNA-to-cDNA kit (Thermo Fisher). The specificity of PCR was verified by performing reactions without RT and melt-curve analysis. Sequences of PCR primers were: Human PIEZO1, (forward) 5'-AGATCTCGCACTCCAT-3', (reverse) 5'-CTCCTTCTCACGAGTCC-3'; Mouse PIEZO1 (forward), 5'- GCTTGCTAGAACTTCACG-3', (reverse) 5'-GTACTCATGCGGGTTG-3'. PCR products were electrophoresed on 2% agarose gels and sequenced to confirm their identity. Real-time PCR was performed on a CFX96 Real-Time PCR detection system (Bio-rad) using SYBR Green Premix Ex Taq II (Takara, Dalian, Liaoning, China). All the 2-ΔΔCT values were normalized using the reference gene β-actin.

### Western Blot

Cells were collected in 1× lysis buffer [20 mM Tris-HCl (pH 7.5), 150 mM NaCl, 1 mM Na_2_EDTA, 1 mM EGTA, 1% Triton, 2.5 mM sodium pyrophosphate, 1 mM beta-glycerophosphate, 1 mM Na_3_VO_4_, 1 µg·ml^-1^ leupeptin] containing protease and phosphatase inhibitor cocktails (Cell Signaling Technology), centrifuged for 10 min at 12,000 *g* at 4°C, and protein concentration quantified by Pierce BCA protein assay kit (Thermo Fisher Scientific). Then, 20 μg protein lysate was run on an 8% gel, transferred to a PVDF membrane, and probed with anti-Piezo1 (Proteintech) and rabbit-anti-GAPDH antibody (Cell Signaling Technology) by standard enhanced chemiluminescence (Millipore). Proteins were visualized using an Image Quant LAS4000 (GE Healthcare) system.

### Data and Statistical Analysis

OriginPro 2018 was used for data and statistical analyses. Average data are presented as means ± SEM; where *n* is the number of independent experiments for a given result and *N* is the number of wells used in multi-well plates. Technical replicates were used to increase our confidence in data from independent experiments. Yoda1 activity after cells were pretreated with TBMS1 was normalized to that of cells pretreated with DMSO. Data subjected to statistical analysis contained at least five independent experiments. Student's t-tests were used for comparisons between two sets of data. For multiple comparisons, one-way ANOVA was used with Tukey's *post hoc* test. *P* < 0.05 was deemed significant. For IC_50_ determination, data were normalized to DMSO, and curves were fitted using the Hill (Origin Pro 2018) equation.

### Materials

Unless stated separately, all chemicals that were commercially available were purchased from Sigma-Aldrich. The natural compound library was purchased from National Institute for the Control of Pharmaceutical and Biological Products (Beijing, China) with purity >98% by high-performance liquid chromatography (HPLC) and stored at −20°C at a stock concentration of 10 mM unless otherwise stated. Fura-2-AM and Fluo-4AM (Thermo Fisher Scientific) were dissolved in DMSO at a concentration of 1 mM. Pluronic acid F127 was in DMSO of 10% w.v^-1^ and stored at room temperature. Yoda1 (Tocris) was prepared as a 10 mM stock solution. The final working concentration was obtained by diluting the stock solution 1:400 in recording solution. Phenylephrine was stored in aqueous solution at 100 mM.

## Results

### TBMS1 Inhibits Endogenous Yoda1-Evoked Piezo1 Channel Activation

**Figure 1:**
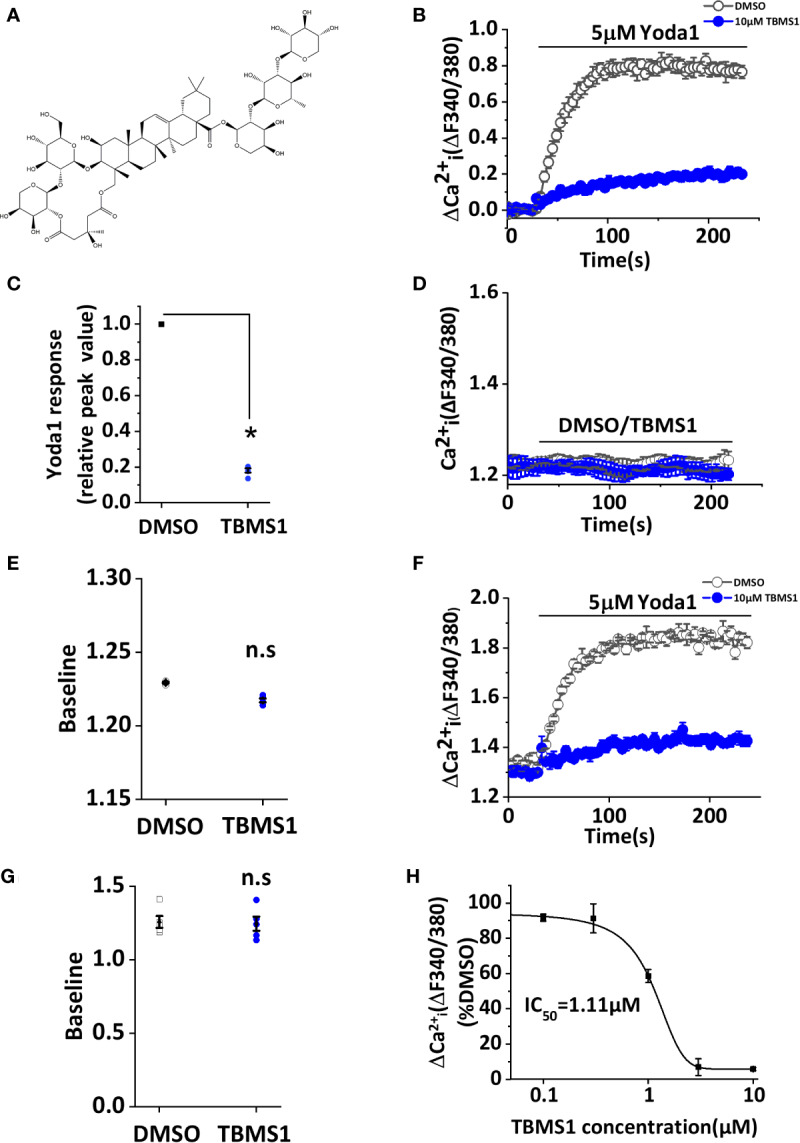
Tubeimoside I (TBMS1) inhibits Yoda1-induced Piezo1 activity. **(A)** Chemical structure of TBMS1. **(B)** Intracellular Ca^2+^ responses of human umbilical vein endothelial cell (HUVECs) to 5 μM Yoda1 after the cells were pretreated for 30 min with 10 μM TBMS1 or DMSO. Error bars indicate SEM (N = 16 each). **(C)** Summary of the results presented in **(B)** expressed as a percentage of the cell response to Yoda1 after cells were pretreated with TBMS1 or DMSO. Error bars indicate SEM (n = 5). **(D)** Intracellular Ca^2+^ measurements in HUVECs when direct application of 10 μM TBMS1 or DMSO only. Error bars indicate SEM (N = 8 each). **(E)** Mean data for the results shown in **(C)** (n = 5). **(F)** Intracellular Ca^2+^ responses of HUVECs to 5 μM Yoda1 after the cells were pretreated for 30 min with 10 μM TBMS1 or DMSO with original baseline. Error bars indicate SEM (N = 16 each). **(G)** Mean data for baseline differences of the results shown in **(F)** (n = 5). **(H)** Mean data of the results shown in **(A)** in which cells were pretreated with the indicated concentrations of TBMS1. Fitting the curve to the Hill equation yields an IC_50_ of 1.11 μM (n=5). *P < 0.05.

We initially screened 92 natural products consisting of different classes of molecules from Traditional Chinese Medicines for effects on Ca^2+^ entry in HUVECs (**Figure S1**). Each product was pre-incubated with HUVECs for 30 min and maintained throughout the recording at a final concentration of 10 μM. During the recordings, Yoda1 at 5 μM was applied to stimulate Piezo1 channel mediated Ca^2+^ entry in the presence of each molecule. With this screen, TBMS1, the structure shown in **Figure 1A**, was found to have inhibitory effect against Yoda1 response (**Figures 1B, C**) but had no effect on baseline, whether application of TBMS1 only or calcium detection after TBMS1 pretreatment (**Figures 1D–G**). Other products with 90% inhibition were not studied because the baseline changed when cells were pretreated with those compounds. TBMS1 had a concentration-dependent inhibitory effect on Yoda1-induced Ca^2+^ entry, with an IC_50_ of 1.11 μM (**Figure 1H**). Together, these data support that TBMS1 is an antagonist of Yoda1-induced Piezo1 channels in endothelial cells.

### TBMS1 May Compete for a Binding Site With Yoda1

**Figure 2:**
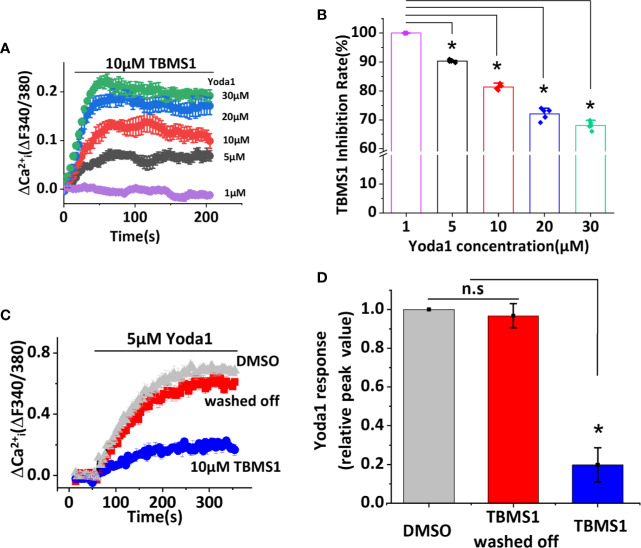
Tubeimoside I (TBMS1) may compete for a binding site with Yoda1 and the inhibitory effect is reversible. **(A)** Intracellular Ca^2+^ measurements with indicated concentrations of Yoda1 after cells were pretreated with 10 μM TBMS1. Error bars indicate SEM (N = 8 each). **(B)** The inhibition rate of TBMS1 with indicated concentrations of Yoda1 measured between 40–60 s after Yoda1 application, expressed as a percentage of the 1 μM Yoda1 response. Error bars indicate SEM (n = 5). *P < 0.05. **(C)** Intracellular Ca^2+^ responses of HUVECs to 5 μM Yoda1 after the cells were pretreated for 15 min with 10 μM TBMS1 (blue trace)/DMSO (grey trace). For the red trace, cells were pretreated for 15 min with 10 μM TBMS1, washed twice with SBS and then intracellular Ca^2+^ measurements were made 15 min after (N = 24 each). **(D)** Summary of the results presented in **(C)** expressed as a percentage of the cell response to Yoda1 after cells were pretreated with DMSO, TBMS1, or washed TBMS1 off. Error bars indicate SEM (n = 5). *P < 0.05.

We have identified TBMS1 as an inhibitor of Yoda1-activated Piezo1 channels, but the mechanism remains unclear. We suspect that higher concentration of Yoda1 may reduce the inhibitory rate of TBMS1. To test this hypothesis we performed the experiments that five concentrations (1, 5, 10, 20, 30 μM) of Yoda1 were used to examine whether TBMS1 has the different effects. Interestingly, with the increase of the concentration of Yoda1, the inhibitory rate of TBMS1 has decreased by 9.65%, 18.61%, 27.94%, 31.89%, respectively (**Figures 2A, B**). The inhibition were reversible (**Figures 2C, D**). The data suggest that there is a competitive effect on Piezo1 channels between TBMS1 and Yoda1.

### TBMS1 Has Selectivity for Piezo1 Channels

**Figure 3:**
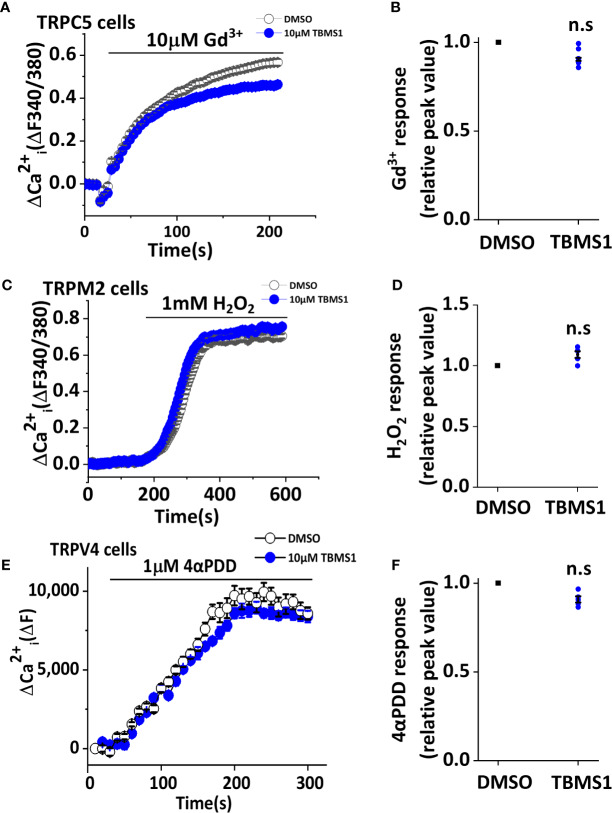
Tubeimoside I (TBMS1) has selectivity of Piezo1. **(A, C)** Intracellular Ca^2+^ measurements in HEK 293T cells overexpressing TRPC5 and TRPM2 channels treated with DMSO or 10 μM TBMS1 and exposed to 10 μM Gd^3+^ **(A)** or 1 mM H_2_O_2_ **(C)**. Error bars indicate SEM (N = 8 each). **(B, D)** Summary of the results shown in **(A, C)**, data were normalized to the peak amplitude values after treatment of cells with DMSO. Error bars representing SEM (n = 5). *P < 0.05. **(E)** Intracellular Ca^2+^ measurements in CHO K1 cells overexpressing TRPV4 channels treated with DMSO or 10 μM TBMS1 and exposed to 1 μM 4αPDD. Error bars indicate SEM (N = 16 each). **(F)** Summary of the results shown in **(E)**, data were normalized to the peak amplitude values after treatment of cells with DMSO. Error bars representing SEM (n = 5). *P < 0.05.

To further investigate whether TBMS1 has a similar inhibitory effect on other channels, we first monitored Ca^2+^ entry through TRPC5 and TRPM2 channels overexpressed in HEK 293T cells. The results showed that pretreatment with 10 μM TBMS1 had no effect on Ca^2+^ entry into HEK293T cells overexpressing TRPC5 activated by 10μM Gd^3+^ (**Figures 3A, B**), or Ca^2+^ entry into HEK293T cells overexpressing TRPM2 channels activated by 1 mM hydrogen peroxide (H_2_O_2_) (**Figures 3C, D**). Another test was taken on mechanical sensitive channel TRPV4 evoked by 1 μM 4α-PDD, TBMS1 had no significant inhibiting effect on this channel (**Figures 3E, F**). The data above suggest that TBMS1 is selective for Piezo1 channels and lacks effect on TRPC5, TRPM2, and TRPV4 channels.

### Inhibition by TBMS1 in Cells Overexpressing Wild-Type Piezo1

**Figure 4:**
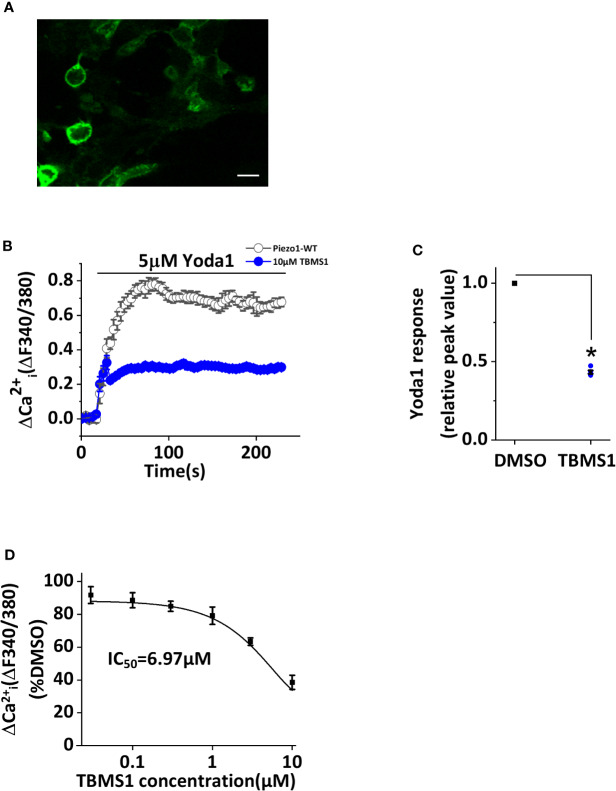
Inhibition by Tubeimoside I (TBMS1) in cells overexpressing wild-type Piezo1. **(A)** HEK 293T cells were transfected to express exogenous Piezo1 tagged with green fluorescent protein (GFP) (scale bar, 100 μm). **(B)** Intracellular Ca^2+^ measurements of transfected cells exposed to 5 μM Yoda1 after cells were pretreated with 10 μM TBMS1 or DMSO. Error bars indicate SEM (N = 24 each). **(C)** Summary of the results in **(B)** expressed as a percentage of the peak amplitude value after cell pretreatment with DMSO. Error bars represent SEM (n = 5). *P < 0.05. **(D)** Mean data of the results shown in **(B)** in which cells were pretreated with the indicated concentrations of TBMS1. Fitting the curve to the Hill equation yields an IC_50_ of 6.97 μM (n=5). *P < 0.05.

To determine whether TBMS1 has inhibitory effects on Piezo1-overexpressing cells, we transfected HEK293T cells with exogenous wild-type Piezo1 tagged with green fluorescent protein (GFP) (**Figure 4A**). Intracellular Ca^2+^ measurements revealed that TBMS1 could inhibit the Yoda1 response (**Figures 4B, C**). TBMS1 also had a concentration-dependent inhibitory effect with an IC_50_ of 6.97μM (**Figure 4D**). Therefore, we conclude that TBMS1 can inhibit the Yoda1 response in HEK293T cells overexpressing wild-type Piezo1.

### TBMS1 Inhibits Yoda1-Induced Piezo1 Activity in MLECs

**Figure 5:**
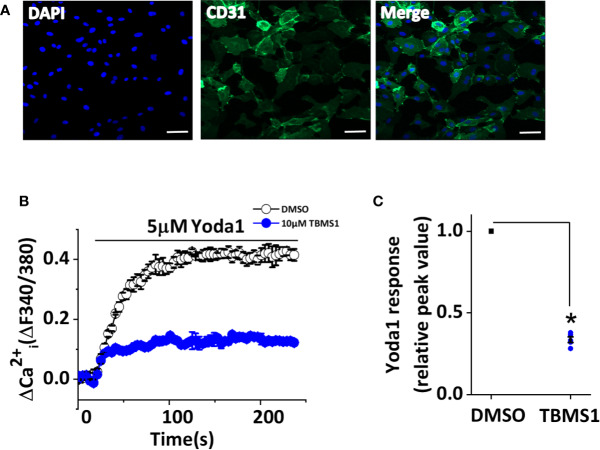
Tubeimoside I (TBMS1) inhibits Piezo1 channels in murine liver endothelial cells (MLECs). **(A)** Extracted murine liver endothelial cells (MLECs) were verified by CD31-labeling of the cell membrane (green) and DAPI-labeling of cell nuclei (blue). Scale bar, 200 μm **(B)** Intracellular Ca^2+^ responses to 5 μM Yoda1 in MLECs after cell pretreatment for 30 min with 10 μM TBMS1 or DMSO. Error bars indicate SEM (N = 16 each). **(C)** Summary of the results in **(B)** expressed as a percentage of the response to Yoda1 in cells pretreated with DMSO only. Error bars indicate SEM (n = 5). *P < 0.05.

Given that TBMS1 can inhibit Yoda1 response in HUVECs, we next explored whether TBMS1 has similar inhibitory effects in native cells. We extracted MLECs and verified them using anti-CD31 antibody (**Figure 5A**). Using intracellular Ca^2+^ measurements, we investigated the function of TBMS1 in MLECs and found that the Yoda1 response was relatively low; unsurprisingly, however, TBMS1 could still suppress the Yoda1 response, reflecting the same effect relative to the responses in HUVECs (**Figures 5B, C**). These results suggest that TBMS1 can inhibit the Yoda1 response in native cells.

### TBMS1 Inhibits Yoda1-Induced Piezo1 Channel Activation in Macrophages

**Figure 6:**
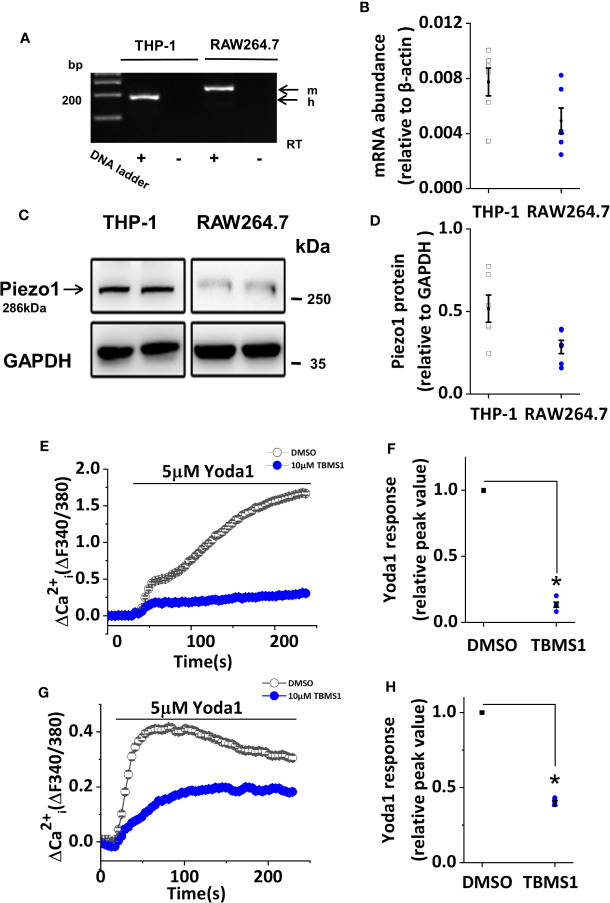
Tubeimoside I (TBMS1) inhibits Yoda1-induced Piezo1 activity in macrophages. **(A)** End-point PCR products obtained with Piezo1 primers for human (h.) THP-1 cells, and mouse (m.) RAW264.7 cells. **(B)** Quantitative real-time PCR data for the experiments shown in **(A)**. **(C)** Western blot of THP-1 cells and RAW264.7 cells with Piezo1 antibody, confirming Piezo1 expression (predicted size, 286 kDa). **(D)** Quantitative protein data for experiments shown in **(C)**. **(E, G)** Intracellular Ca^2+^ measurements in THP-1 cells and RAW264.7 cells exposed to 5 μM Yoda1 after cell pretreatment with 10 μM TBMS1 or DMSO. Error bars indicate SEM (N = 24 each). **(F, H)** Summary of the results shown in **(E, G)** expressed a percentage of the peak amplitude value relative to the response to 5 μM Yoda1 in cells pretreated with DMSO alone. Error bars represent SEM (n = 5). *P < 0.05.

To explore the effect of TBMS1 on Piezo1 channels more broadly, we studied THP-1 and RAW264.7 cells. mRNA encoding Piezo1 could be clearly detected in THP-1 and RAW264.7 cells (**Figures 6A, B**), and these results were mirrored by western blots to monitor protein levels (**Figures 6C, D**). TBMS1 inhibited Yoda1-evoked Ca^2+^ entry into THP-1 cells (**Figures 6E, F**), and similar results were acquired in endothelial cells, whereas a smaller inhibitory effect of TBMS1 was observed in RAW264.7 cells (**Figures 6G, H**). All together, these results manifest that TBMS1 also inhibits Yoda1-induced channel activity in macrophages and has no cell-type specificity.

### TBMS1 Inhibits Yoda1-Induced Relaxation of Aorta

**Figure 7:**
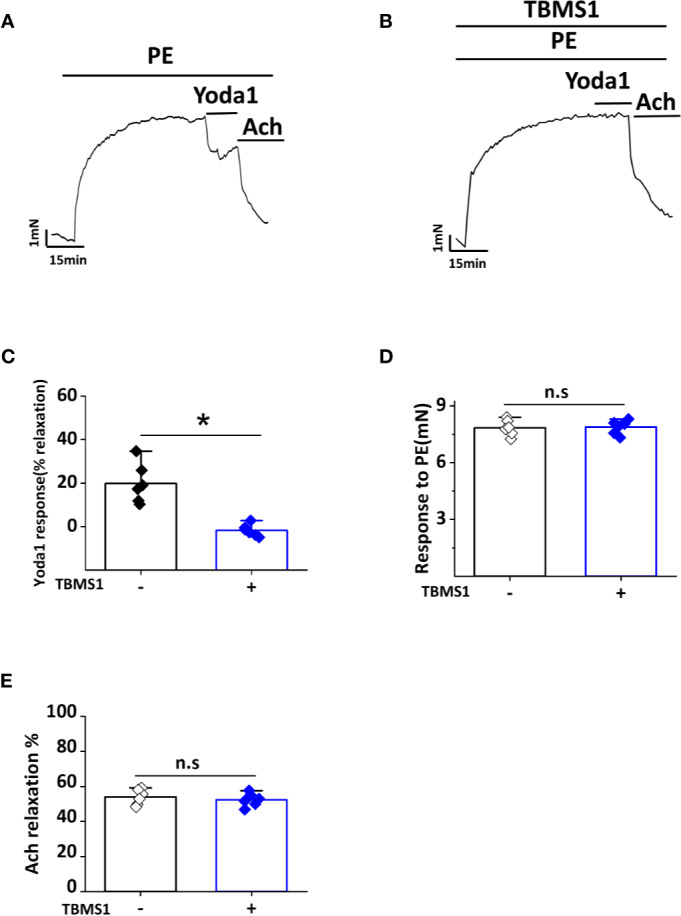
Tubeimoside I (TBMS1) inhibits Yoda1-induced dilation in aorta. **(A)** Aorta pre-constricted with PE and exposed to 5 μM Yoda1. **(B)** As shown in **(A)** but after aorta pretreatment for 30 min with 10 μM TBMS1. **(C)** Summary of the results in **(A, B)** expressed as the percentage relaxation evoked by Yoda1. Each data point represents a value from an independent experiment. Error bars represent SEM (n = 6). *P < 0.05. **(D, E)** Analysis of the PE and Ach response in the presence of TBMS1 revealed no significant inhibition. Error bars represent SEM (n = 6). *P < 0.05.

A previous study showed that Yoda1 caused endothelium-dependent relaxation in mouse thoracic aorta by stimulating NO production *via* the endothelium (Evans et al., 2018). To determine whether TBMS1 inhibits Yoda1-induced relaxation, we pre-incubated aortic rings with 10 μM TBMS1 for 30 min. Yoda1-induced relaxation was strongly restrained by TBMS1 (**Figures 7A–C**). Analysis of the PE and Ach response in the presence of TBMS1 revealed no significant inhibition (**Figures 7D, E**). The data suggest that TBMS1 is a potent inhibitor of Yoda1-induced aortic relaxation, mediated by interruption of Piezo1 channel activity induced by Yoda1.

## Discussion

The results of this study provide insight into compounds from Chinese Medicine and could facilitate the development of new tools for investigating Piezo1 channel function. Through this research, we identified TBMS1 as an inhibitor of Yoda1-induced Piezo1 channel activity. We confirmed our hypothesis by confirming the blocking activity of TBMS1, its competing effect on various concentrations of Yoda1, its specificity for Piezo1 channels, and its effects on physiological responses to isometric tension. Similar effects were observed in MLECs and macrophages. This study reveals that Yoda1 inhibitors exist in TCM and deepen our understanding of the vascular pharmacology of Piezo1 channels.

One of the interesting results is that the inhibitory effects of TBMS1 declined with the increase of Yoda1 concentration (**Figure 2A**), indicating that there is a competing effect, which is reversible (**Figure 2C**) on Yoda1-induced Piezo1 channel activity. The inhibitory effects were also investigated in various cell lines, including macrophages. However, one divergence from the data was that the potency of TBMS1 in cells overexpressing wild-type Piezo1 was not as great in HUVECs. We speculate that on one hand, there might be conformational changes upon Piezo1 overexpression which altering the channel activity. On the other hand, endothelial cells have proved to have higher opening basal state when stimulated by Yoda1 as previously reported (Rode et al., 2017; Evans et al., 2018). Similarly, TBMS1 exhibited partial antagonism in RAW264.7 cells, in contrast to greater inhibition in THP-1 cells, reflecting that Yoda1 response was dependent upon Piezo1 and proportional to its expression level and thus less sensitive to channel inhibition.

TBMS1 strongly inhibited Yoda1 in both HUVECs and in aorta (**Figures 1A** and **7A**). The data indicate that TBMS1 is more efficacious than a previously reported compound (Evans et al., 2018). Similarly, although the inhibitory effect of TBMS1 on PE-induced contractions of aortic rings has not affected (**Figure 7D**) but Yoda1 induced relaxation has significantly reduced, indicating TBMS1 directly inhibiting Piezo1 channel activity or by other unknown mechanisms. It is also possible that TBMS1 acts on Piezo1 in the smooth muscle cells of the vessel, which inhibiting contraction in part.

Bolbostemma, a natural plant tuber, is widely used in the pharmaceutical industry (Yang et al., 2016; Chen et al., 2018; He et al., 2018). TBMS1 extracted from Bolbostemma attenuates inflammation as well as depression of angiogenesis (Jia et al., 2015; Wu et al., 2018; Islam et al., 2019), and it can inhibit the growth of vascular sprouts from the aortic wall in Matrigel (Gu et al., 2016). However, few studies have examined the mechanism by which TBMS1 inhibits angiogenesis. Based on the inhibiting effect of TBMS1 in HUVECs, we speculate that the anti-angiogenic effect of TBMS1 may be related to the Piezo1 channel, but this notion requires further experimental confirmation.

This study revealed a specific chemical inhibitor of Piezo1 channels based on Yoda1. TBMS1 is not perfect, but, nonetheless, it is a new tool that can effectively antagonize Yoda1-induced Piezo1 channel activity and will therefore be useful for pharmacological studies of Piezo1. Although it is not clear whether activating or inhibiting this channel is beneficial, harnessing the therapeutic potential of Piezo1 will be necessary in the future to expand our pharmacological and physiological conceptions of this protein. An improved understanding of the interactions between Piezo1 channels and small molecules will be an indispensable aspect to understanding the biology of Piezo1. In conclusion, our data demonstrate that TBMS1 can strongly inhibit the Yoda1 response, and that this compound is selective for Piezo1 channels. These observations reveal the existence of Yoda1 inhibitors and improve our understanding of vascular pharmacology related to Piezo1 channels.

## Data Availability Statement

All datasets generated for this study are included in the article/**Supplementary Material**.

## Ethics Statement

The animal study was reviewed and approved by Guangzhou University of Chinese Medicine Animal Ethics Committee.

## Author Contributions

SL conducted and analyzed the experiments. XP, WC, and BD helped in initial screening experiments. YH, LZ, and YN performed data analysis. JL conceived the idea for the project, generated research funds, and led and coordinated the study. SL and JL designed the study and wrote the paper. All authors reviewed the results, commented on the manuscript, and approved the final version of the manuscript.

## Funding

The study was supported by the National Natural Science Foundation of China (NSFC 81770453) and special fund from the Open Project of Key Laboratory of Basic Research of Traditional Chinese Medicine in Shandong Province.

## Conflict of Interest

The authors declare that the research was conducted in the absence of any commercial or financial relationships that could be construed as a potential conflict of interest.

## Abbreviations

TBMS1, tubeimoside I; HUVECs, human umbilical vein endothelial cells; TRPC5, transient receptor potential canonical 5; TRPM2, transient receptor potential melastatin 2; TRPV4, transient receptor potential vanilloid 4; CHO, Chinese hamster ovary; PE, phenylephrine; MLECs, murine liver endothelial cells; 4α-PDD, 4α-phorbol-didecanoate.
