# Supplementary material for: Tubeimoside I Antagonizes Yoda1-Evoked Piezo1 Channel Activation
Source: Front Pharmacol. 2020 May 25;11:768. doi: 10.3389/fphar.2020.00768 (PMC7261832; doi:10.3389/fphar.2020.00768)
Supplement: Supplementary file 1 [file DataSheet_1.pdf]

Figure S1 Chemical structures and inhibitory effects of 92 natural products from Traditional Chinese Medicine used in the screen.

	Product Name	Formula	Inhibition Ratio
1	Tubeimoside I	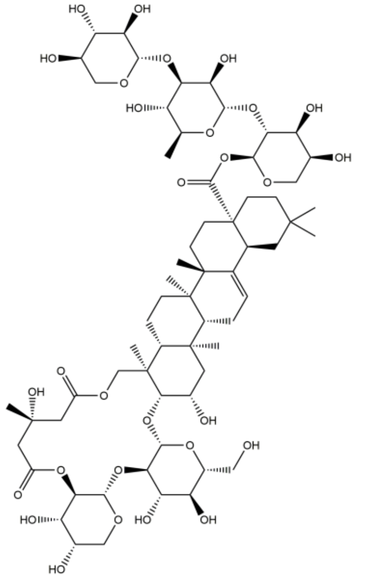	90%
2	Neomangiferin	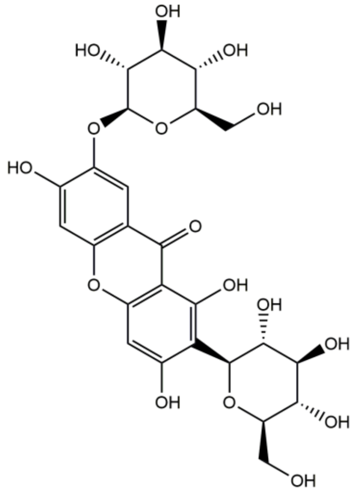	90%
3	Glabridin	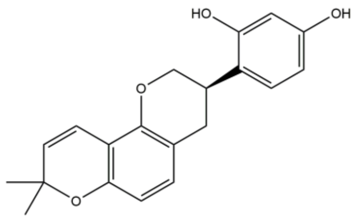	90%

4	Mangiferin	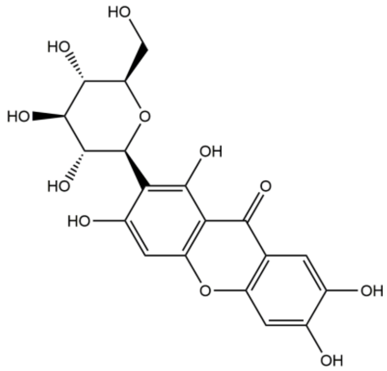	90%
5	Arecoline HBr	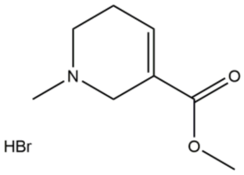	80%
6	Alpha-Mangostin	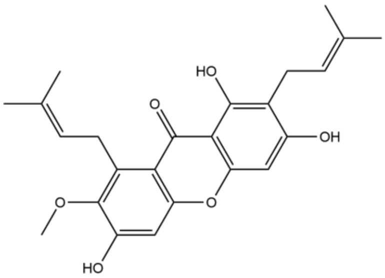	70%
7	Artemisinin	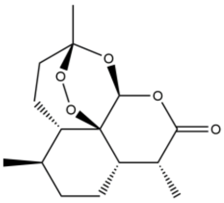	60%
8	Xylose	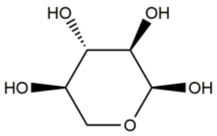	60%
9	Bakuchiol	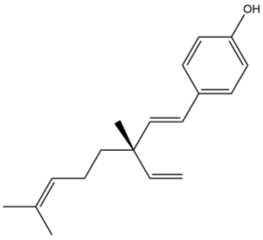	60%
10	Rutaecarpine	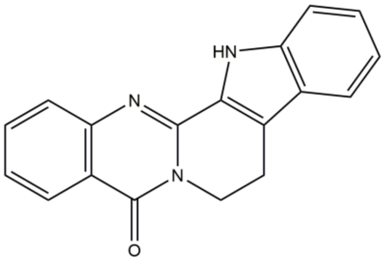	50%

11	Schizandrol A	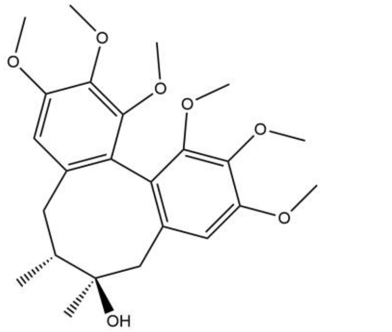	50%
12	Echinacoside	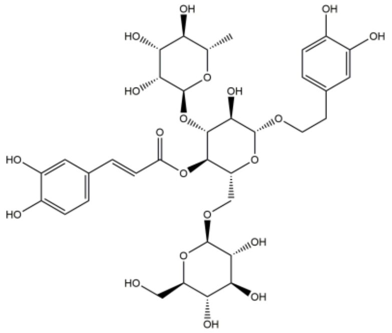	45%
13	Vanillylacetone	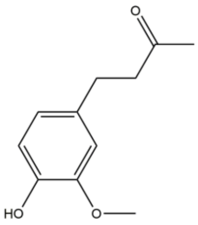	40%
14	Erythritol	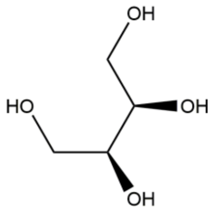	40%
15	Sinomenine hydrochloride	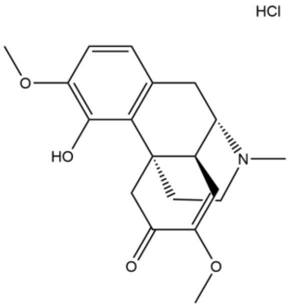	40%
16	Magnolol	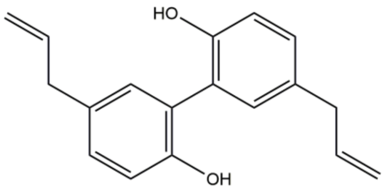	40%

17	Salidroside	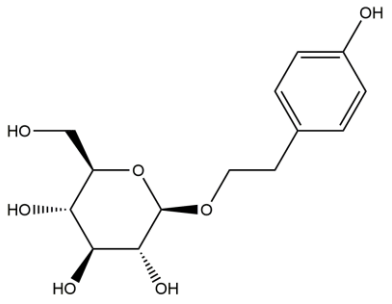	33.30%
18	Ligustrazine hydrochloride	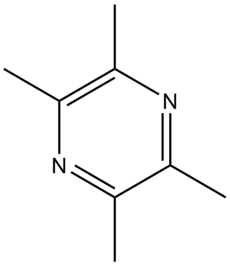 HCl	33.30%
19	cis-Anethole	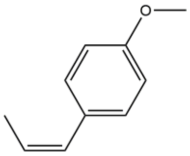	30%
20	Imperatorin	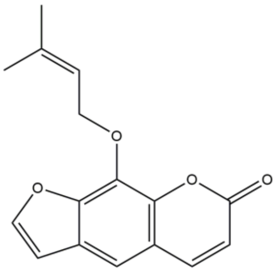	30%
21	Angelic anhydride	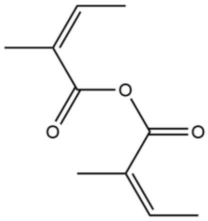	28%
22	Docetaxel	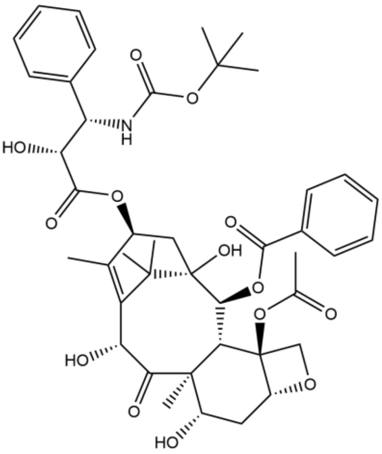	25%

23	Lycorine hydrochloride	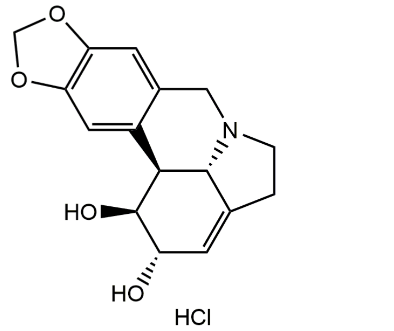	24%
24	Tetrahydropiperine	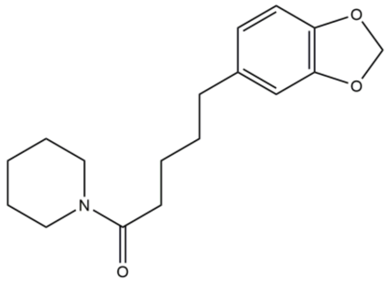	23%
25	Sophocarpine	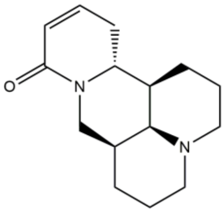	20%
26	Betaine	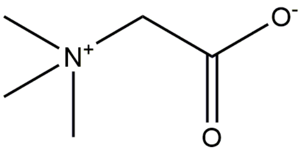	20%
27	Ginsenoside Re	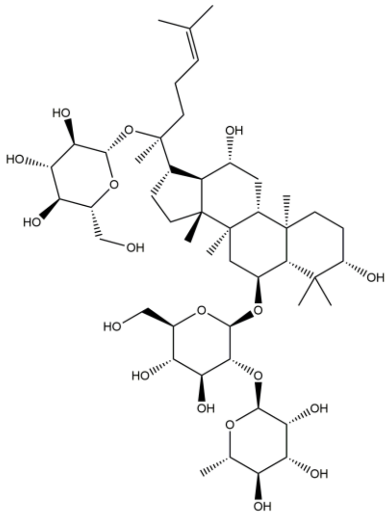	20%

28	Mogroside V	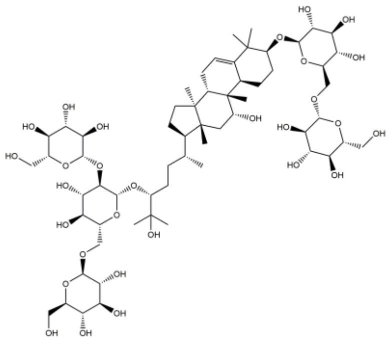	20%
29	Citral	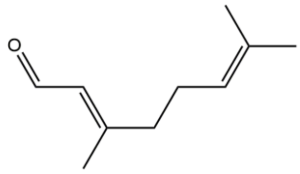	20%
30	Genipin	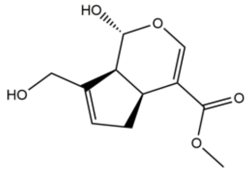	18%
31	Trigonelline Hydrochloride	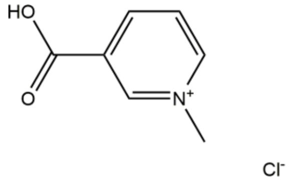	18%
32	Tiglic acid	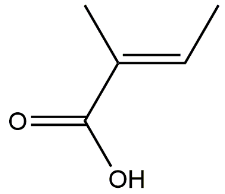	16%
33	Angelic acid	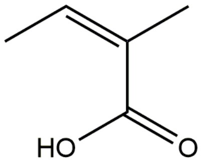	16%

34	Stevioside	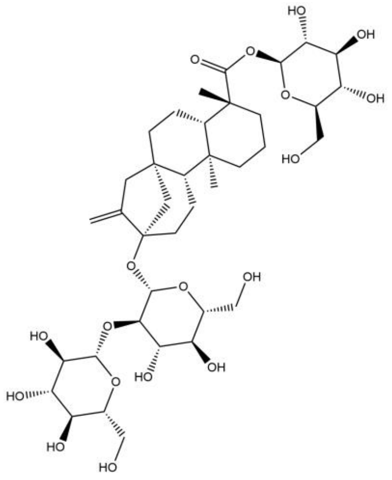	16%
35	Geniposide	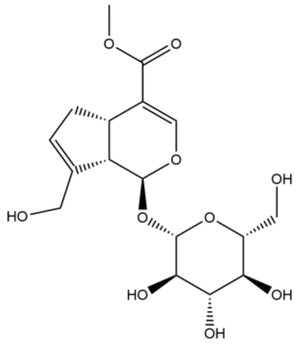	15%
36	Picroside II	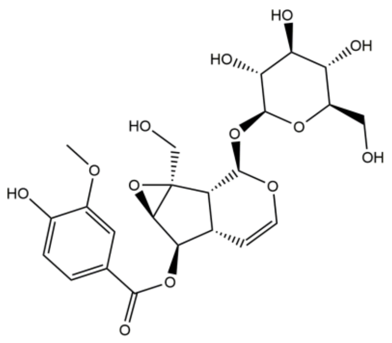	15%
37	Gentiopicroside	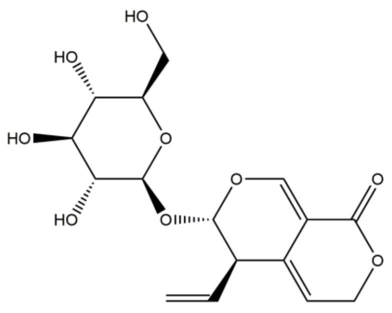	15%

38	Picroside I	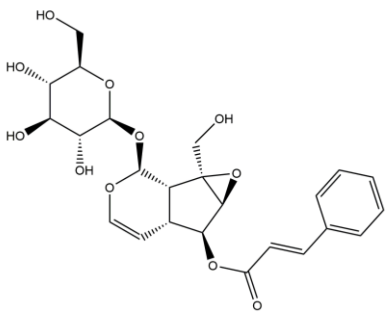	15%
39	Dehydroandrographolide	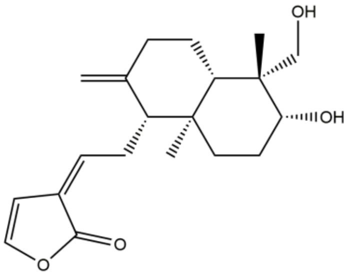	15%
40	Carvacrol	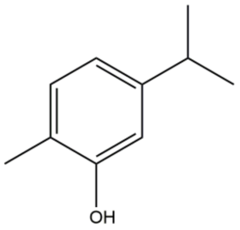	14%
41	Dihydromyricetin	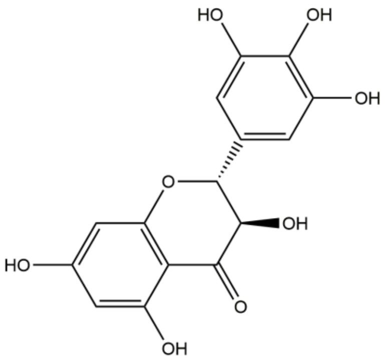	10%
42	Norcantharidin	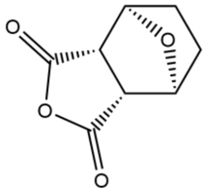	10%
43	Sophoricoside	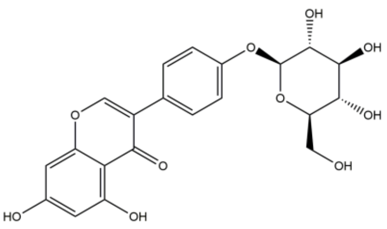	10%

44	Ginkgolide C	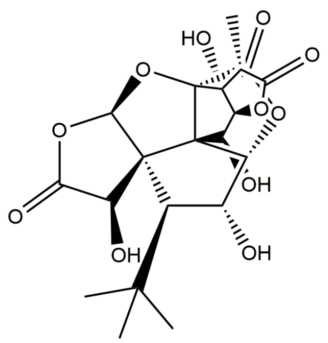	10%
45	Neferine	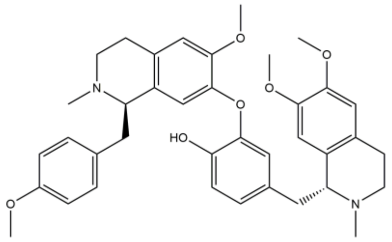	10%
46	Coumarin	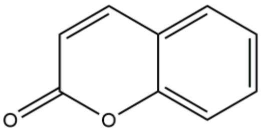	9%
47	Celastrol	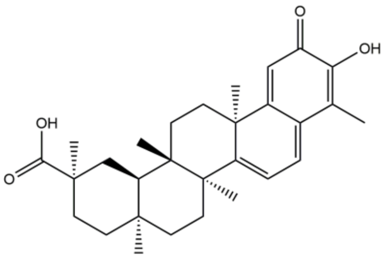	8%
48	Patchouli alcohol	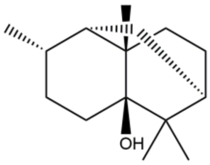	8%
49	4-Methoxysalicylic acid	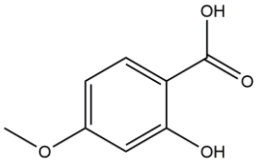	8%
50	Methyl gallate	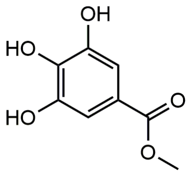	7%

51	Paclitaxel	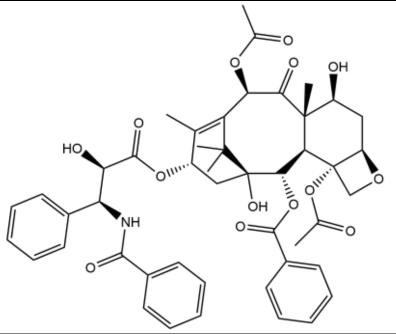	5%
52	Hordenine	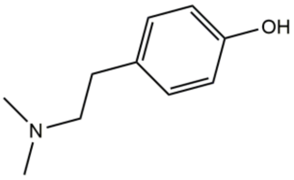	5%
53	Capsaicin	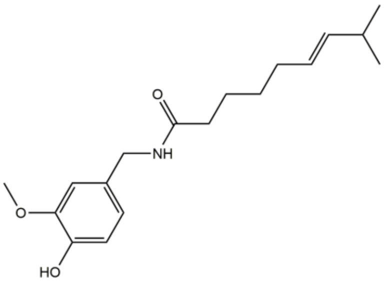	5%
54	Rutin	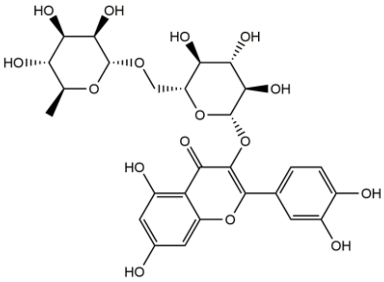	3%
55	Gastrodin	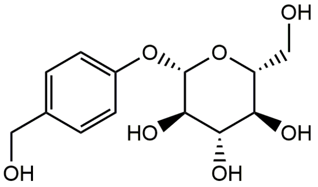	3%
56	Ammonium Glycyrrhizinate	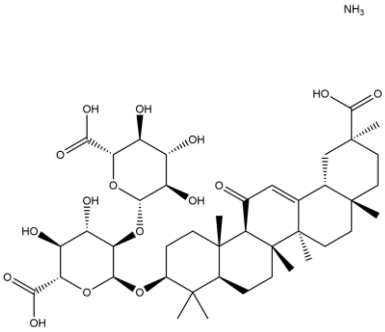	3%

57	Orsellinic acid ethyl ester	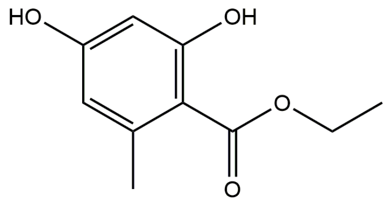	3%
58	Triptolide (PG490)	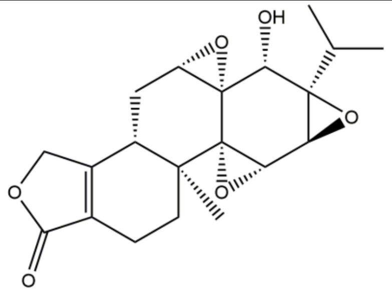	3%
59	Paeoniflorin	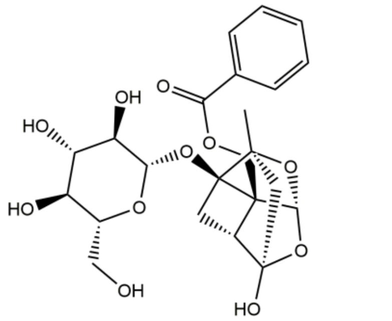	2%
60	Cinchonine(LA40221)	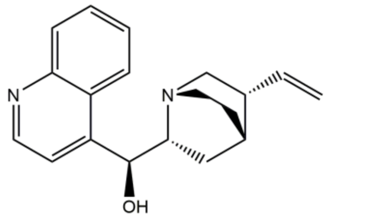	2%
61	Genistin (Genistoside)	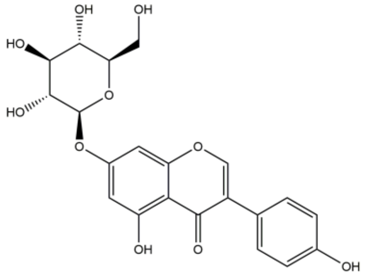	2%
62	Sclareol	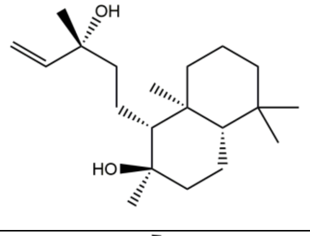	0%
63	Sclareolide	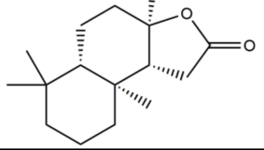	0%

64	Silibinin	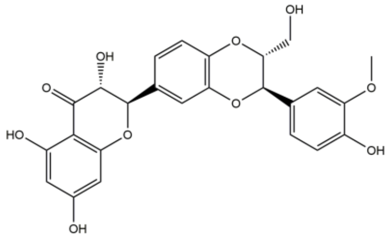	0%
65	Sinomenine	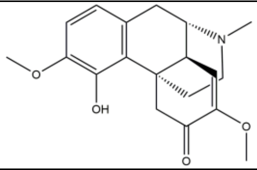	0%
66	Tangeretin	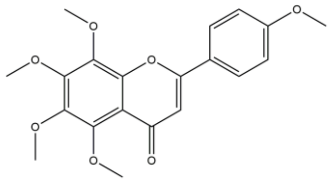	0%
67	Taxifolin (Dihydroquercetin)	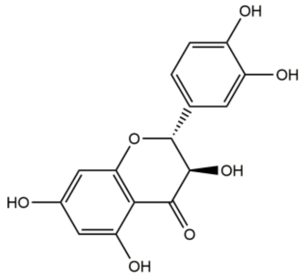	0%
68	Biochanin A	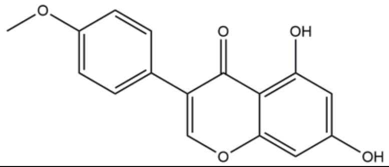	0%
69	Dioscin	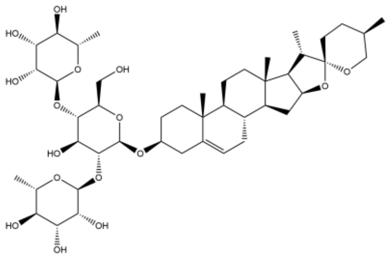	0%
70	Diosmetin	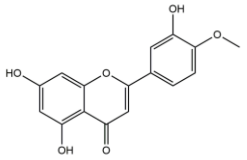	0%
71	Lappaconite HBr	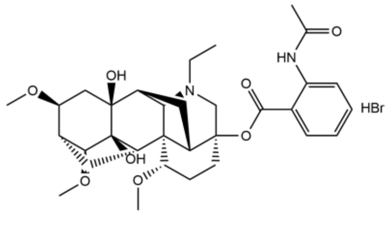	0%

72	Naringin Dihydrochalcone	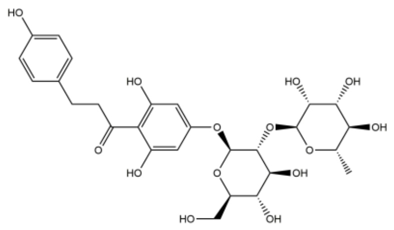	0%
73	Polydatin	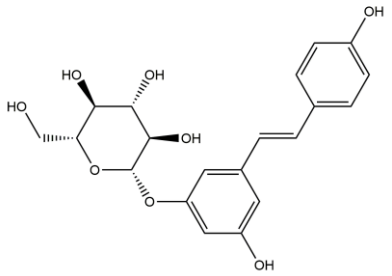	0%
74	Sesamin	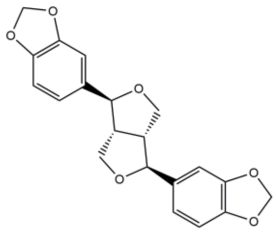	0%
75	Naringenin	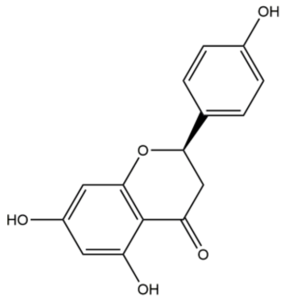	0%
76	Curcumol	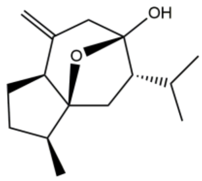	0%
77	Geniposidic acid	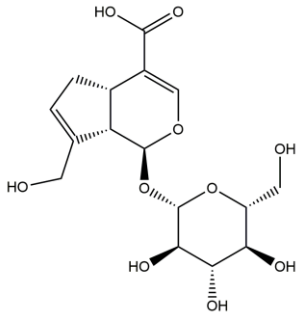	0%

78	Astragaloside A	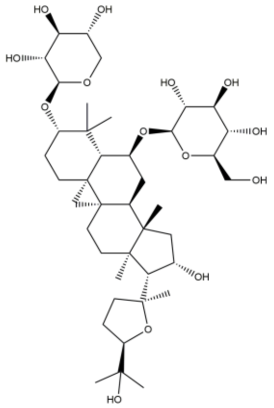	0%
79	Apocynin	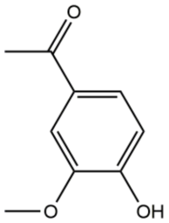	0%
80	Cephalomannine	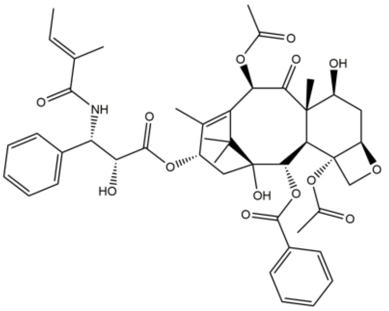	0%
81	Cinnamic acid	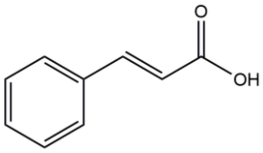	0%
82	Cinnamaldehyde	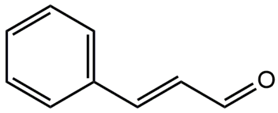	0%
83	10,11-Dimethoxystrychnine	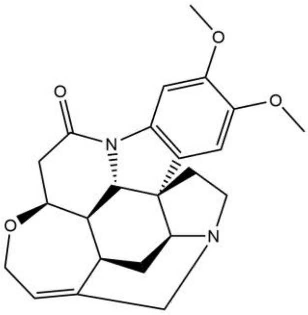	0%
84	Succinic acid	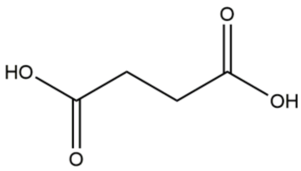	0%

85	Guggulsterone E&Z	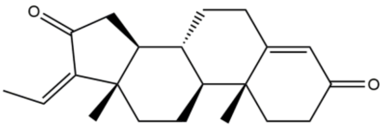	0%
86	Rebaudioside A	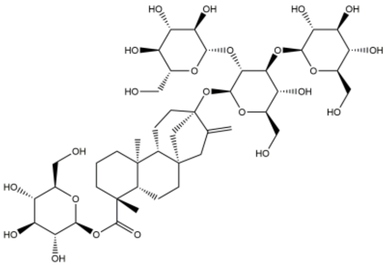	0%
87	Monocrotaline	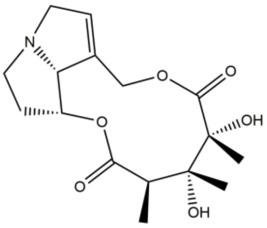	0%
88	Taurocholic acid sodium salt hydrate	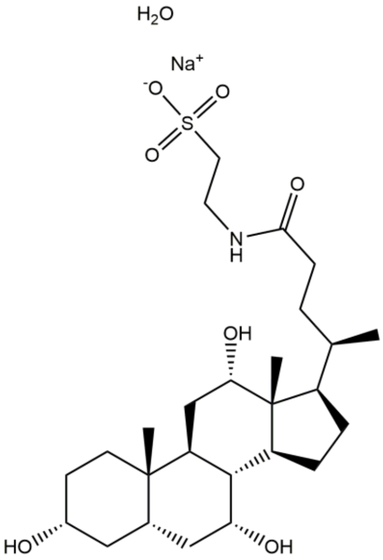	0%
89	Raffinose	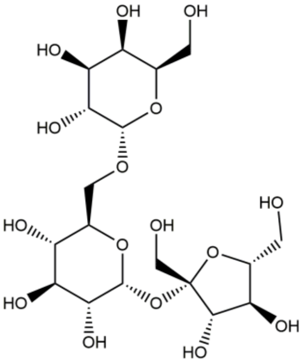	0%

90	Thymol	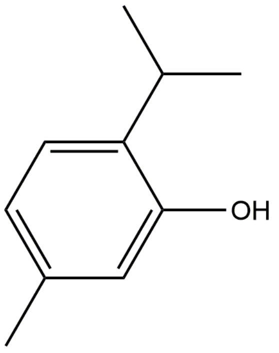	0%
91	7-Methoxycoumarin	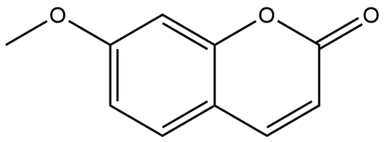	0%
92	Benzyl cinnamate	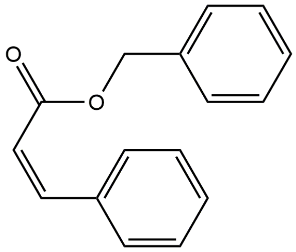	0%
